# Controlled Synthesis of Diphosphine-Protected Gold Cluster Cations Using Magnetron Sputtering Method

**DOI:** 10.3390/molecules27041330

**Published:** 2022-02-16

**Authors:** Lewei Wang, Tsubasa Omoda, Kiichirou Koyasu, Tatsuya Tsukuda

**Affiliations:** 1 Department of Chemistry, School of Science, The University of Tokyo, Bunkyo-ku, Tokyo 113-0033, Japan; wanglewei@chem.s.u-tokyo.ac.jp (L.W.); t.omoda@chem.s.u-tokyo.ac.jp (T.O.); kkoyasu@chem.s.u-tokyo.ac.jp (K.K.); 2 Elements Strategy Initiative for Catalysts and Batteries (ESICB), Kyoto University, Katsura, Kyoto 615-8520, Japan

**Keywords:** ligand-protected gold cluster, magnetron sputtering, atomically precise synthesis, polyethylene glycol

## Abstract

We demonstrated, for the first time, atomically precise synthesis of gold cluster cations by magnetron sputtering of a gold target onto a polyethylene glycol (PEG) solution of 1,3-bis(diphenylphosphino)propane (Ph_2_PCH_2_CH_2_CH_2_PPh_2_, dppp). UV-vis absorption spectroscopy and electrospray ionization mass spectrometry revealed the formation of cationic species, such as [Au(dppp)*_n_*]^+^ (*n* = 1, 2), [Au_2_(dppp)*_n_*]^2+^ (*n* = 3, 4), [Au_6_(dppp)*_n_*]^2+^ (*n* = 3, 4), and [Au_11_(dppp)_5_]^3+^. The formation of [Au(dppp)_2_]^+^ was ascribed to ionization of Au(dppp)_2_ by the reaction with PEG, based on its low ionization energy, theoretically predicted, mass spectrometric detection of deprotonated anions of PEG. We proposed that [Au(dppp)_2_]^+^ cations thus formed are involved as key components in the formation of the cluster cations.

## 1. Introduction

Ligand-protected gold clusters with atomically-defined sizes constitute a distinct class of nanomaterials, showing size-specific optical and catalytic properties [1,2,3,4,5,6]. Their conventional synthetic method is based on the chemical reduction of Au(I)-ligand complexes. Key processes involved in the cluster production include the competition between nucleation/cluster growth and passivation with the ligands. However, the actual processes are much more complicated, such that the choice of precursor complexes is a critical factor that determines the products. For example, chemical reduction of Au(PPh_3_)L (L = Cl, NO_3_, SR) yielded [Au_11_(PPh_3_)_8_Cl_2_]^+^ [7], [Au_9_(PPh_3_)_8_]^3+^ [8], and Au_13_(PPh_3_)_8_(SR)_3_ [9], respectively. As such, the atomically-controlled, targeted synthesis of ligand-protected Au clusters remains challenging.

A simple approach for targeted synthesis is to control the kinetics of the nucleation/aggregation of naked Au atoms in the presence of the ligands. One efficient way to supply naked Au atoms is through the magnetron sputtering method [10,11]. Since the first report of the production of silver and iron nanoparticles in silicon oil by Wagner [12], various metal nanoparticles have been efficiently prepared by magnetron sputtering onto liquids containing surfactants and ligands [13,14] or ionic liquids [15,16,17,18,19]. Au clusters smaller than 2 nm have been successfully obtained utilizing thiols as protective ligands [20].

**Scheme 1:**
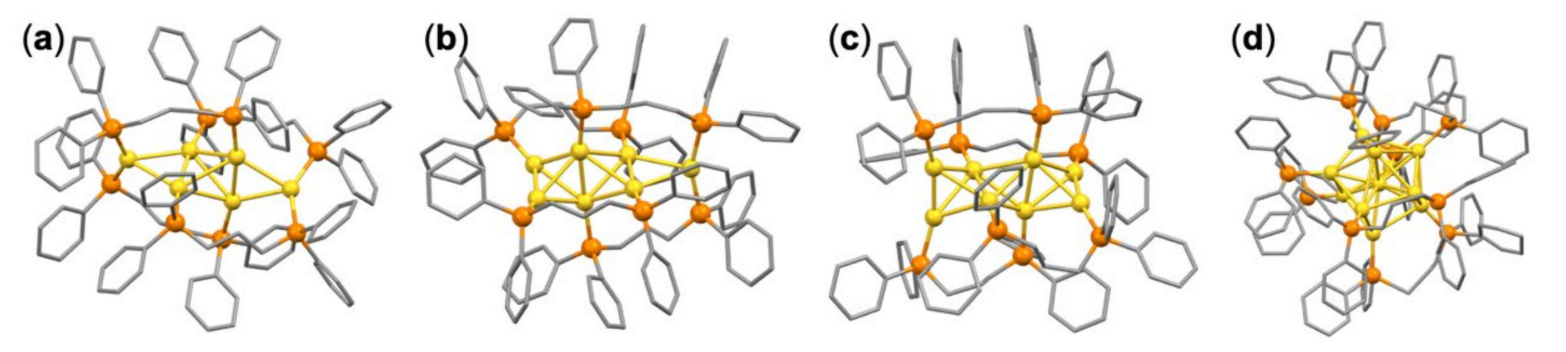
Crystal structures of (**a**) [Au_6_(dppp)_4_]^2+^ [21], (**b**) [Au_7_(dppp)_4_]^3+^ [22], (**c**) [Au_8_(dppp)_4_]^2+^ [23] and (**d**) [Au_11_(dppp)_5_]^3+^ [24]. Color codes: yellow (Au); orange (P). Phenyl groups and alkyl chains are shown by gray sticks. H atoms are omitted.

In the present study, we attempted to synthesize Au clusters by magnetron sputtering of an Au target onto a polyethylene glycol (PEG) solution containing neutral ligands. We chose a diphosphine ligand, dppp (Ph_2_PCH_2_CH_2_CH_2_PPh_2_), as a neutral ligand, since candidate reaction products could be predicted from the literature. A series of cationic forms of dppp-protected Au clusters have been synthesized by chemical methods, e.g., [Au_6_(dppp)_4_]^2+^ [21], [Au_7_(dppp)_4_]^3+^ [22], [Au_8_(dppp)_4_]^2+^ [23], [Au_8_(dppp)_4_Cl_2_]^2+^ [23], and [Au_11_(dppp)_5_]^3+^ [24]. Single crystal structures in Scheme 1 revealed that the Au_6–8_ cores of [Au_6_(dppp)_4_]^2+^, [Au_7_(dppp)_4_]^3+^, and [Au_8_(dppp)_4_]^2+^ possessed [core + *exo*] type structures with prolate shapes, while the Au_11_ core of [Au_11_(dppp)_5_]^3+^ had an icosahedral-like shape. The dppp-protected Au clusters formed by magnetron sputtering under different concentrations of dppp were characterized by mass spectrometry and optical spectroscopy. Unexpectedly, cluster cations with known and unknown compositions were formed. The formation process of these cluster cations was then discussed, with a focus on ionization mechanisms.

## 2. Results and Discussion

### 2.1. Formation and Characterization of Au Clusters Synthesized by Magnetron Sputtering

The dppp-protected Au clusters were prepared using an auto fine coater designed to prepare specimens for scanning electron microscopy by coating them with the conductive layer (see the materials and methods part for more detail). In short, Au atoms and clusters generated by the magnetron sputtering were deposited onto the PEG solutions of dppp with a concentration of *x* mM. The sputtering current and sputtering time of the auto fine coater were set to 10 mA and 600 s, respectively, so that the total amount of Au atoms deposited was comparable for all the samples. The dppp-protected Au clusters produced were washed with the aqueous solution of NaNO_3_ to collect them by precipitation as nitrate salts. The Au clusters thus obtained are hereafter referred to as Au-dppp-*x*.

**Figure 1:**
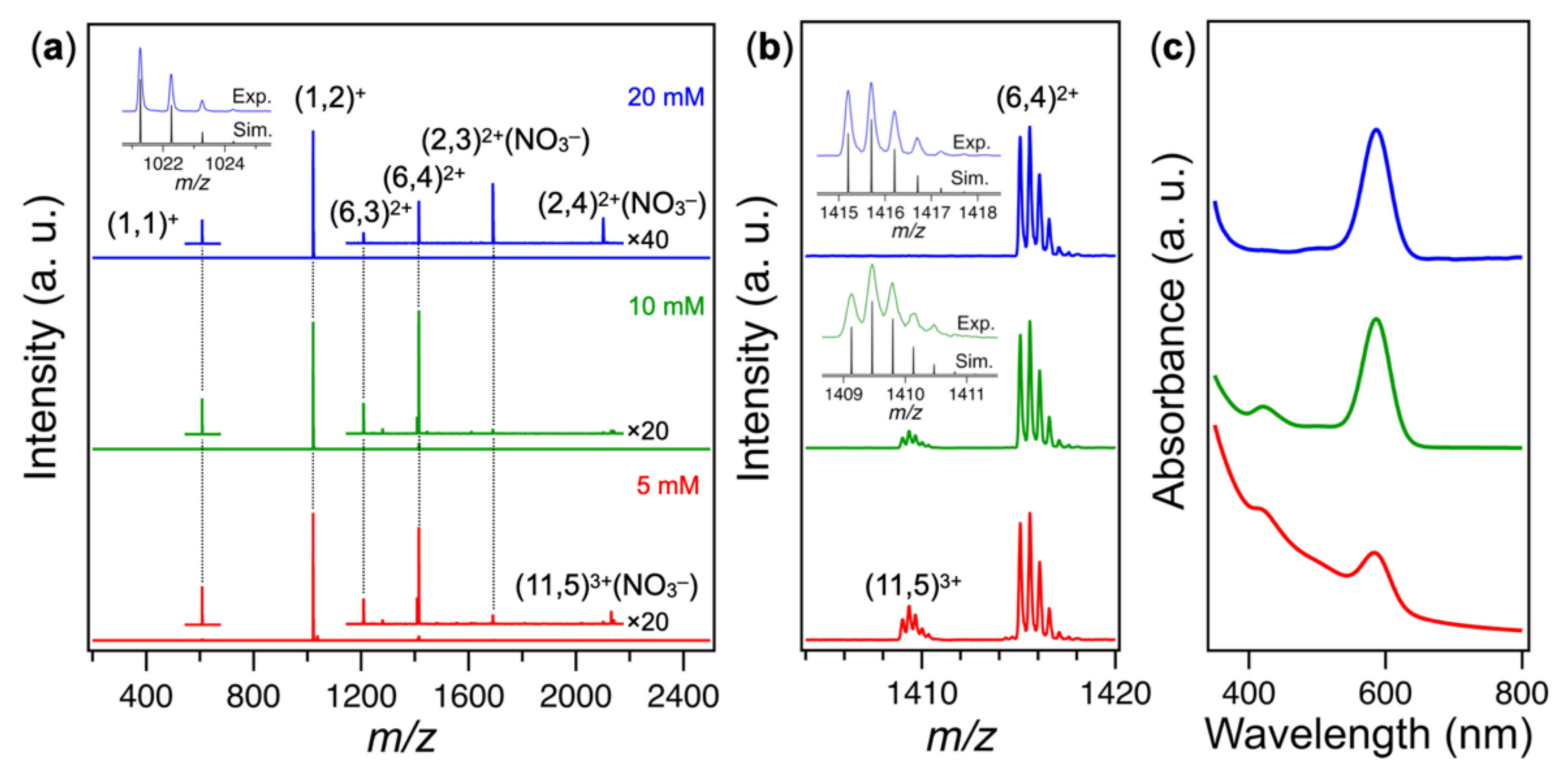
(**a**) Positive-mode ESI mass spectra of Au-dppp-*x*: *x* = 20 (blue), 10 (green), and 5 (red). Notation (*a*, *b*)*^q^*^+^ represents [Au*_a_*(dppp)*_b_*]*^q^*^+^. Inset shows the experimental and theoretical isotope pattern of (1, 2)^+^. (**b**) Enlarged view of panel (**a**) in the *m*/*z* range of 1404–1420. Insets show the experimental and theoretical isotope patterns of (6, 4)^2+^ and (11, 5)^3+^. (**c**) UV-vis absorption spectra of the acetonitrile solutions of Au-dppp-*x*.

The chemical compositions of the cationic clusters in the Au-dppp-*x* samples were investigated by electrospray ionization (ESI) mass spectrometry as a function of the dppp concentration. Figure 1a shows the ESI mass spectra of Au-dppp-*x* (*x* = 5, 10, and 20). The detailed assignment is shown in Figure S1. All the mass spectra were dominated by a series of isotope peaks assigned to [Au(dppp)_2_]^+^, indicating that [Au(dppp)_2_]^+^ was formed as a dominant cation by the magnetron sputtering onto the PEG solution of dppp. In addition to the other monomeric cations [Au(dppp)]^+^, larger Au cluster cations were detected in the mass spectra, whose relative populations changed with the dppp concentrations. The mass spectrum of Au-dppp-20 (blue trace, Figure 1a) exhibited a series of mass peaks for the dimer [Au_2_(dppp)_3,4_]^2+^ in the form of NO_3_^−^ adducts together with the hexamer [Au_6_(dppp)_3,4_]^2+^. In the mass spectrum of Au-dppp-10, [Au_2_(dppp)_3,4_]^2+^ disappeared (green trace, Figure 1a) and [Au_11_(dppp)_5_]^3+^ appeared instead (green trace, Figure 1b). The relative population of [Au_11_(dppp)_5_]^3+^ with respect to [Au_6_(dppp)_3,4_]^2+^ increased further in the mass spectrum of Au-dppp-5 (red trace, Figure 1b). Other gold cluster cations, such as [Au_7_(dppp)_4_]^3+^ [22] and [Au_8_(dppp)_4_]^2+^ [23], shown in Scheme 1, were not detected. These results indicated the growth in the sequence of [Au(dppp)*_n_*]^+^ (*n* = 1, 2) → [Au_2_(dppp)*_n_*]^2+^ (*n* = 3, 4) → [Au_6_(dppp)*_n_*]^2+^ (*n* = 3, 4) → [Au_11_(dppp)_5_]^3+^ with decrease in dppp concentration.

Ultraviolet-visible (UV-vis) absorption spectra of the Au-dppp-*x* (*x* = 20, 10, and 5) samples are shown in Figure 1c. The absorption peaks of [Au(dppp)*_n_*]^+^ and [Au_2_(dppp)*_n_*]^2+^ appeared outside the wavelength range. The spectrum of Au-dppp-20 (blue trace, Figure 1c) was dominated by the peak at 584 nm assigned to [Au_6_(dppp)_4_]^2+^ with the known structure (Scheme 1a) [25], consistent with the mass spectrum. Nonappreciable contribution from [Au_6_(dppp)_3_]^2+^ was ascribed to the lower population relative to [Au_6_(dppp)_4_]^2+^ and/or similar optical spectrum with [Au_6_(dppp)_4_]^2+^, although the geometric structure was unknown. In the spectrum of Au-dppp-10 (green trace, Figure 1c), another peak developed at 420 nm, which agreed with that of the known magic cluster [Au_11_(dppp)_5_]^3+^ (Scheme 1d) [26]. In the spectrum of Au-dppp-5 (red trace, Figure 1c), we observed an emergence of a continuous band that increased toward the shorter wavelength due to larger, atomically-polydisperse Au clusters. These larger Au clusters were most likely neutral in the charge state since they were not detected by the ESI mass spectra (red trace, Figure 1a). These results demonstrated that atomically defined magic Au clusters such as [Au_6_(dppp)_3,4_]^2+^ and [Au_11_(dppp)_5_]^3+^ could be synthesized by magnetron sputtering onto the dppp solution with controlled concentration.

**Figure 2:**
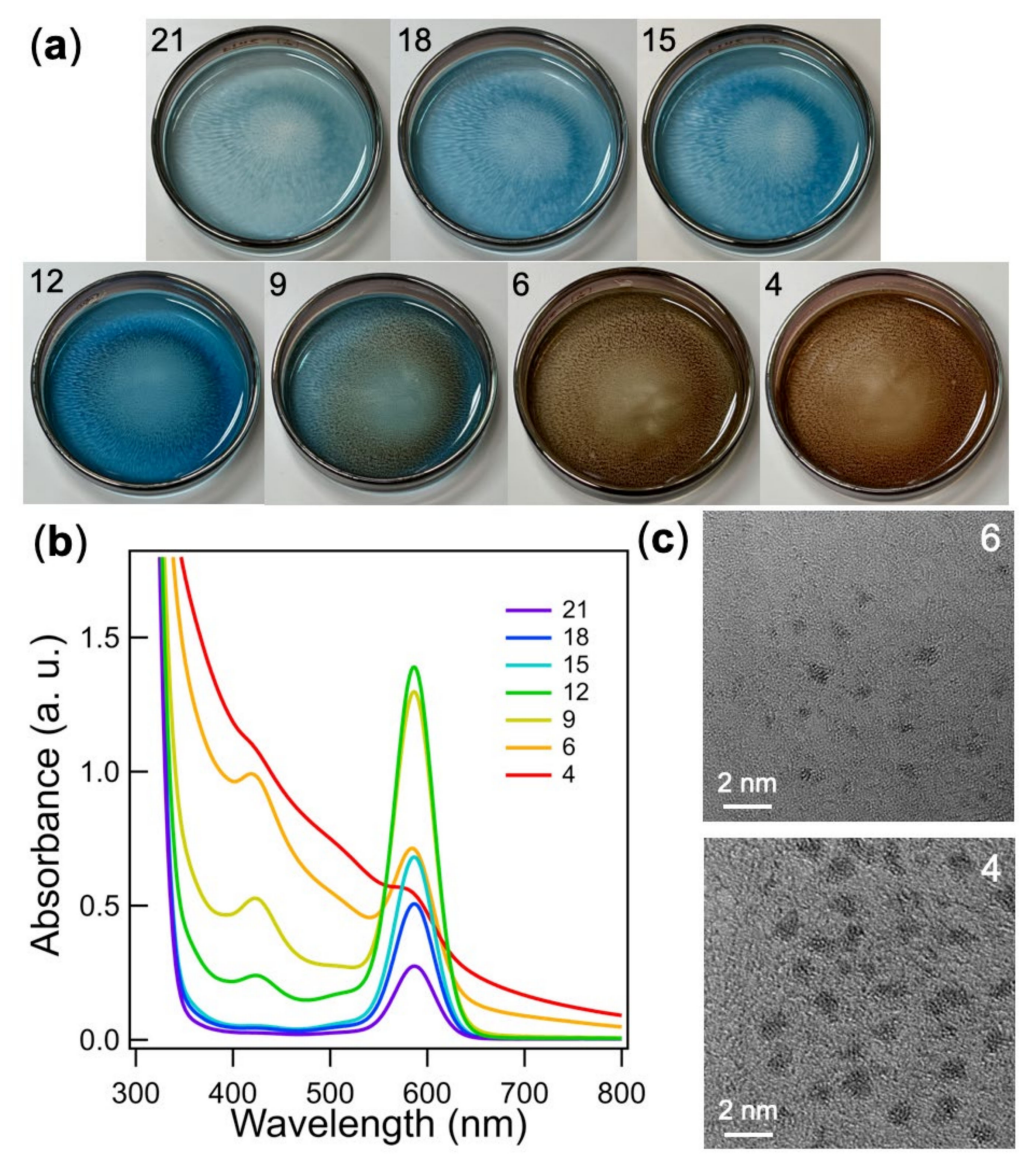
(**a**) The photo images of the PEG solutions with *x* = 21, 18, 15, 12, 9, 6, and 4 after magnetron sputtering. (**b**) UV-vis absorption spectra of the acetonitrile solutions of Au-dppp-*x* (*x* = 4–21). (**c**) Representative TEM images of Au-dppp*-x* (*x* = 4, 6).

In order to obtain a whole picture of the cluster growth process after the magnetron sputtering, we quantitatively compared the UV-vis absorption spectra of another set of the Au-dppp-*x* samples. Figure 2a,b show photo images and the corresponding absorption spectra of the PEG solutions after magnetron sputtering as a function of *x*, respectively. The color of the PEG solution was pale blue at *x* = 21 due to the absorption peak at 584 nm originated from [Au_6_(dppp)_4_]^2+^ [25]. The blue color became more intense with the reduction of *x* to 15, in accordance with increase of the peak intensity at 584 nm. This behavior indicated that the production of [Au_6_(dppp)_3,4_]^2+^ was promoted by the reduction of dppp concentration. At *x* = 12, the brownish ring was observed in the photo image and another peak at 420 nm emerged. This trend grew more pronounced at *x* = 9. This result indicated that the growth to [Au_11_(dppp)_5_]^3+^ was promoted by further reduction of *x*. The ring patterns implied that the Au_6_ and Au_11_ clusters were distributed nonhomogeneously in the unstirred, viscous PEG solutions. At *x* = 6 and 4, the PEG solutions became brownish, but did not exhibit an absorption peak at ~520 nm due to the localized surface plasmon resonance. This result indicated the formation of Au clusters smaller than 2 nm in Au-dppp-6 and Au-dppp-4, which was confirmed by transmission electron microscopy (TEM) (Figure 2c). The above results implied that the yield of specific clusters could be maximized by optimizing the dppp concentration.

### 2.2. Mechanism of the Formation of Au Cluster Cations by Magnetron Sputtering

**Figure 3:**
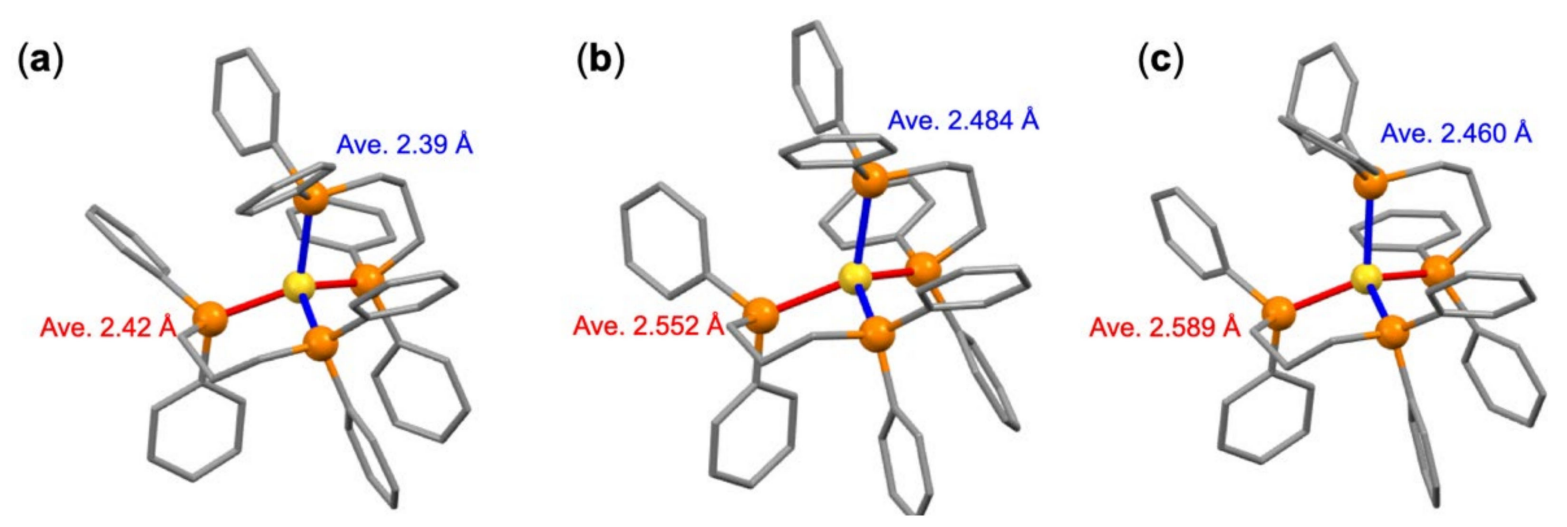
(**a**) Crystal structure of [Au(dppp)_2_]^+^ [29]. DFT-optimized structures of (**b**) [Au(dppp)_2_]^+^ and (**c**) Au(dppp)_2_ using BP86 functional. Color codes: yellow (Au); orange (P). Phenyl groups and alkyl chains are shown as gray sticks. H atoms are omitted. The lengths represent the average values of long (red) and short (blue) Au–P bonds of individual dppp ligands.

In our magnetron sputtering source, major species deposited onto the PEG solution were neutral Au atoms rather than Au^+^ ions, since the Au target placed on the electrode was biased to negative voltage with respect to the stage for the PEG solution. Thus, the initial step was the capture of Au atoms by the dppp ligands dissolved in the PEG. Nevertheless, we detected the cationic species [Au(dppp)_2_]^+^ as the major ionic species (Figure 1a), indicating that Au-dppp complexes were spontaneously ionized in the PEG. Similar phenomena were observed by introducing the vapor of Au(0) atoms into the solution of phosphine ligands [27,28]. For example, [Au(PPh_3_)_2_]^+^ was obtained by evaporating Au atoms into the solution of PPh_3_ and KSCN [27]. To gain insight into the driving force of the formation of cationic clusters, we conducted density functional theory (DFT) calculations on the ionization energy of Au(dppp)_2_. The geometric structures of [Au(dppp)_2_]^+^ and Au(dppp)_2_ were optimized using BP86 functional, starting from a crystal structure of [Au(dppp)_2_]^+^ in which two dppp ligands coordinated to an Au atom tetrahedrally (Figure 3a [29]). The DFT optimized structures of [Au(dppp)_2_]^+^ and Au(dppp)_2_ are shown in Figure 3b,c. The optimized structure of [Au(dppp)_2_]^+^ qualitatively reproduced the crystal structure [29]; the average Au–P bond length calculated was longer than the experimental length determined by ~5%. The adiabatic ionization energy of Au(dppp)_2_ calculated is listed in Table 1 together with those of Au and Cs atoms. The ionization energy of Au(dppp)_2_ was significantly smaller than that of Au and grew smaller than that of Cs. The significant reduction of the ionization energy of Au(dppp)_2_ was ascribed to much stronger bonding energies of dppp to Au^+^ than to Au.

It is known that alkali metal atoms like Cs are ionized by reactions with hydroxy groups of organic compounds. We hypothesized that [Au(dppp)_2_] with lower ionization energy is ionized by the reaction with PEG according to the following equation:

2Au(dppp)_2_ + 2H(OCH_2_CH_2_)*_n_*OH → 2[Au(dppp)_2_]^+^ + 2[H(OCH_2_CH_2_)*_n_*O]^−^ + H_2_. (1)

**Figure 4:**
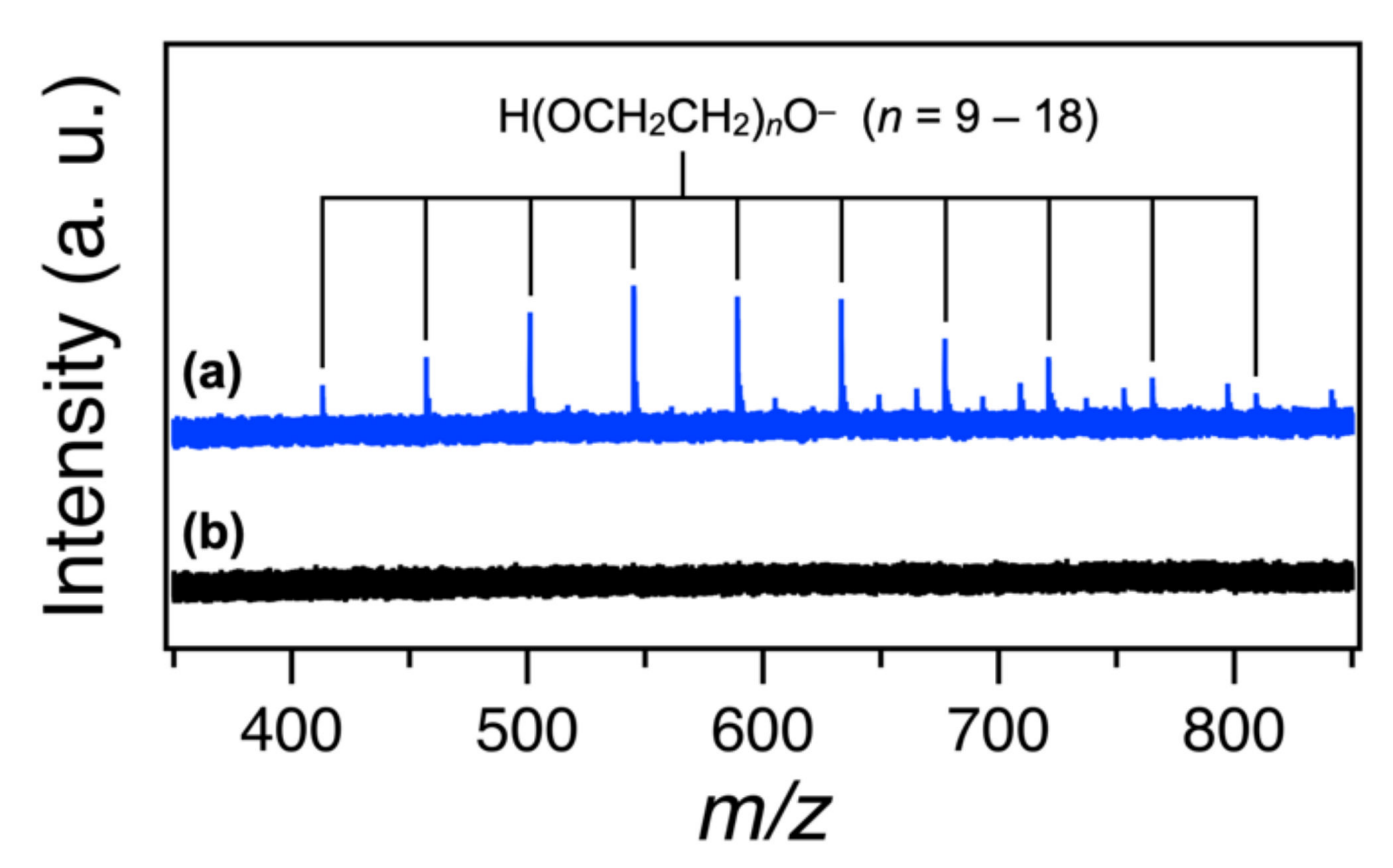
Negative-mode ESI mass spectra of the methanol solutions of PEG solutions (**a**) with and (**b**) without magnetron sputtering.

Formally, Au(dppp)_2_ is ionized by the abstraction of an electron by the proton formed by the deprotonation of PEG. This hypothesis is supported by the detection of deprotonated anions of PEG in the negative-mode ESI-MS of the PEG solution after magnetron sputtering (Figure 4). Namely, the mixing of sputtered PEG solution with aqueous solution of NaNO_3_ resulted in an anion exchange process from [H(OCH_2_CH_2_)*_n_*O]^−^ to NO_3_^−^, leading to the precipitation of the Au-dppp clusters.

## 3. Materials and Methods

### 3.1. Materials

All chemicals used were commercially available. Gold target plate (diameter = 57 mm, thickness = 0.1 mm, purity = 99.99%) was purchased from Furuuchi Chemical Corporation (Tokyo, Japan). Polyethylene glycol (PEG-600, average molecular weight = 600), sodium nitrate (NaNO_3_, > 99%), hexane, toluene, dichloromethane, diethyl ether and methanol were purchased from FUJIFILM Wako Pure Chemical Corporation (Osaka, Japan). 1,3-Bis(diphenylphosphino)propane (Ph_2_PCH_2_CH_2_CH_2_PPh_2_, dppp, > 98%) was purchased from Tokyo Chemical Industry Co., Ltd. (Tokyo, Japan). Deionized water used was Milli-Q grade (>18 MΩ). PEG-600 was stirred in a flask under vacuum at a speed of 800 rpm at 90 °C for at least 3 h to remove dissolved water and gases before use.

### 3.2. Preparation of Au Clusters by Sputtered Deposition

The magnetron sputtering of the gold target was carried out on a JEC-3000FC auto fine coater (JEOL Ltd., Tokyo, Japan). An argon gas cylinder was connected to the auto fine coater with a stagnation pressure of 0.04 MPa. First, a PEG solution of dppp (5 mL) with a concentration of 4–21 mM was poured into a Petri dish with a diameter of 60 or 55 mm for quantified spectral measurement or mass spectral analysis, respectively. The distance between the Au target and the solution surface in the Petri dish was set at 30 mm. Then, the sputtering chamber was evacuated for at least 30 min by pumping with a rotary pump (100 L·h^–1^). The chamber was flushed by Ar gas at least 3 times to sweep out the remaining air. Finally, the sputtering process was initiated within a small flow of Ar reaching 7 Pa. The sputtering current was set to 10 mA. After 300 s of sputtering, the PEG solution was taken out of the chamber and stirred homogeneously. Then, the PEG solution was sputtered again using the same procedure; the total sputtering time was 600 s.

### 3.3. Purification of Au Cluster Products

The magnetron-sputtered PEG solution was mixed with a saturated NaNO_3_ aqueous solution to reach a total volume of 50 mL. The liquid mixture was centrifuged at 3500 rpm for 1 h. After removal of the supernatant, the precipitate was then washed by deionized water, hexane, toluene, and diethyl ether successively to remove NaNO_3_ and excessive dppp.

### 3.4. Characterization

Ultraviolet-visible (UV-vis) absorption spectra of the products in acetonitrile were recorded on a V-670 spectrometer (JASCO Corporation, Tokyo, Japan) with a quartz cell. Electrospray ionization (ESI) mass spectra of the products were obtained on a JMS-T100LC time-of-flight mass spectrometer (JEOL Ltd.). The specimens were prepared by dissolving the samples in acetonitrile with the concentration of 0.1 mg·mL^–1^. ESI mass spectra of PEG were obtained by dissolving pure PEG and magnetron sputtered PEG solution in methanol and the ratio of PEG and methanol was 1:9 (*v*/*v*).

Transmission electron microscopy (TEM) images of the products were acquired on a JEM-ARM200F NEOARM Atomic Resolution Analytical Electron Microscope (JEOL Ltd., Tokyo, Japan). The specimens were prepared by drop-casting methanol solutions of the samples onto a thin carbon-coated copper grid (SHR-C075, Okenshoji, Tokyo, Japan).

### 3.5. Theoretical Calculation

All density functional theory (DFT) calculations were conducted using the Turbomole software package [31]. The initial structure of the tetrahedral Au(dppp)_2_ was constructed from the reported crystal data [29]. Structural optimization of Au(dppp)_2_, [Au(dppp)_2_]^+^, dppp and Au were conducted with the BP86 functional and the def-SV(P) basis sets with def-ecp effective core potentials for Au. The vibrational frequencies for the optimized structures of Au(dppp)_2_ and [Au(dppp)_2_]^+^ were calculated to confirm that those structures were located at local minima of the potential energy surfaces.

## 4. Conclusions

In this work, [Au_6_(dppp)_4_]^2+^ and [Au_11_(dppp)_5_]^3+^ cluster cations were successfully synthesized by supplying magnetron-sputtered Au atoms into a PEG solution of dppp. The relative population of [Au_6_(dppp)_4_]^2+^ and [Au_11_(dppp)_5_]^3+^ could be tuned by controlling the kinetic balance between cluster growth and passivation by dppp. We proposed that Au(dppp)_2_ was ionized by the reaction with PEG based on its small ionization energy theoretically predicted and mass spectrometric detection of deprotonated anions of PEG. The results suggested that the [Au(dppp)_2_]^+^ cations thus formed were key building components of the cluster cations observed. This work provided a simple synthetic route of ligand-protected Au clusters alternative to the conventional chemical synthesis.

## Figures and Tables

**Table 1:** Calculated ionization energies in eV.

Targets	Theoretical	Experimental
Au(dppp)_2_	2.90	–
Au	9.73	9.23 [30]
Cs	4.04	3.89 [30]

## Data Availability

Raw data are available from the authors upon request.

## Supplementary Materials

The following is available online, Figure S1: Expanded views of positive-mode ESI mass spectra of (a) Au-dppp-20 and (b) Au-dppp-5. The theoretical isotope pattern of each species is shown as black bars.

## Sample Availability

Samples of the compounds are not available from the authors.
