# Supplementary material for: Controlled Synthesis of Diphosphine-Protected Gold Cluster Cations Using Magnetron Sputtering Method
Source: Molecules. 2022 Feb 16;27(4):1330. doi: 10.3390/molecules27041330 (PMC8879177; doi:10.3390/molecules27041330)
Supplement: Supplementary file 1 [file molecules-27-01330-s001.zip › molecules-1583856-supplementary.pdf]

molecules

Controlled Synthesis of Diphosphine-Protected Gold Cluster Cations Using Magnetron Sputtering Method

Lewei Wang [1], Tsubasa Omoda [1], Kiichirou Koyasu [1,2] and Tatsuya Tsukuda [1,2,*]

SUPPLYMENTARY MATERIALS

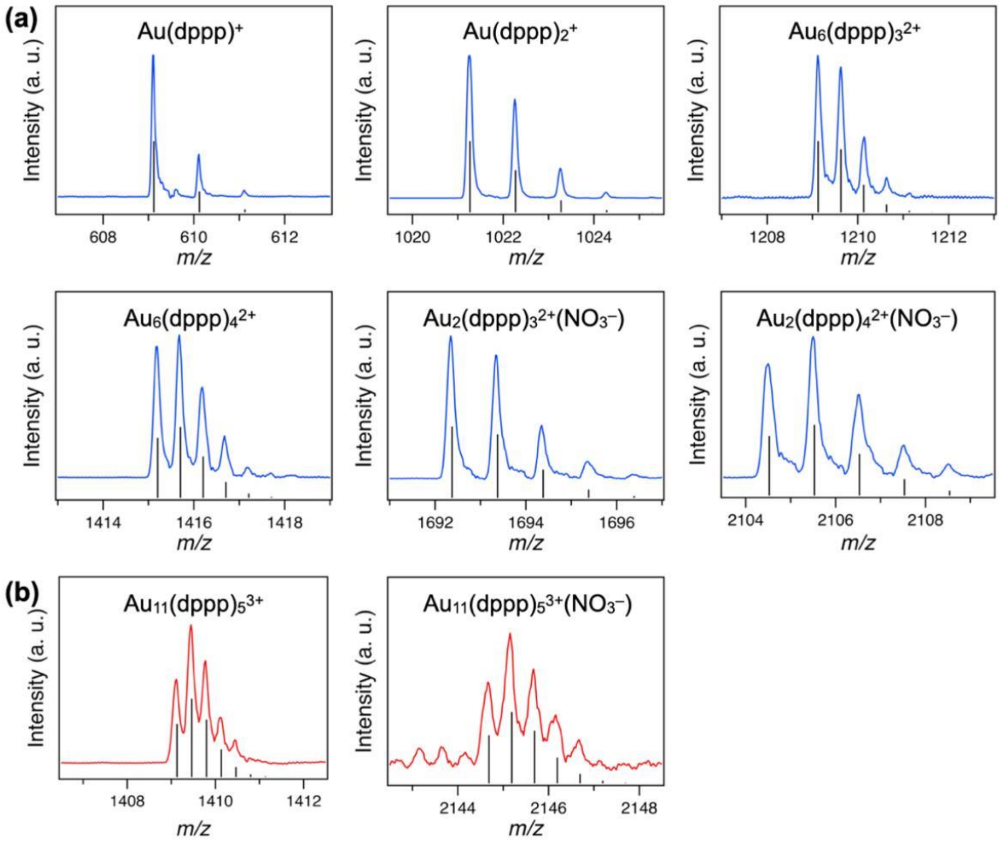

Figure S1. Expanded views of positive-mode ESI mass spectra of (**a**) Au-dppp-20 and (**b**) Au-dppp-5. The theoretical isotope pattern of each species is shown as black bars.
